# A Glycine Riboswitch in *Streptococcus pyogenes* Controls Expression of a Sodium:Alanine Symporter Family Protein Gene

**DOI:** 10.3389/fmicb.2018.00200

**Published:** 2018-02-20

**Authors:** Afsaneh Khani, Nicole Popp, Bernd Kreikemeyer, Nadja Patenge

**Affiliations:** Institute of Medical Microbiology, Virology and Hygiene, University Medicine Rostock, Rostock, Germany

**Keywords:** glycine, riboswitch, *Streptococcus pyogenes*, *cis*-regulatory element, amino acid riboswitch, regulatory RNAs, transcription genetic

## Abstract

Regulatory RNAs play important roles in the control of bacterial gene expression. In this study, we investigated gene expression regulation by a putative glycine riboswitch located in the 5′-untranslated region of a sodium:alanine symporter family (SAF) protein gene in the group A *Streptococcus pyogenes* serotype M49 strain 591. Glycine-dependent gene expression mediated by riboswitch activity was studied using a luciferase reporter gene system. Maximal reporter gene expression was observed in the absence of glycine and in the presence of low glycine concentrations. Differences in glycine-dependent gene expression were not based on differential promoter activity. Expression of the SAF protein gene and the downstream putative cation efflux protein gene was investigated in wild-type bacteria by RT-qPCR transcript analyses. During growth in the presence of glycine (≥1 mM), expression of the genes were downregulated. Northern blot analyses revealed premature transcription termination in the presence of high glycine concentrations. Growth in the presence of 0.1 mM glycine led to the production of a full-length transcript. Furthermore, stability of the SAF protein gene transcript was drastically reduced in the presence of glycine. We conclude that the putative glycine riboswitch in *S. pyogenes* serotype M49 strain 591 represses expression of the SAF protein gene and the downstream putative cation efflux protein gene in the presence of high glycine concentrations. Sequence and secondary structure comparisons indicated that the streptococcal riboswitch belongs to the class of tandem aptamer glycine riboswitches.

## Introduction

Bacterial riboswitches are *cis*-regulatory elements found in the 5′-untranslated regions (UTRs) of mRNAs. Regulation of gene expression by riboswitches is widespread in bacteria. In general, aptamer structures within the riboswitch recognize small molecule ligands and interact with an expression platform. Ligand binding promotes formation of an exclusive conformation and leads to the regulation of downstream genes. Repression of gene expression is achieved by transcription termination, inhibition of translation via sequestration of the ribosome binding site, or mRNA processing. Binding of ligands which induce gene expression, allows transcription elongation or translation initiation. Riboswitches from different classes recognize a large range of cellular compounds, including coenzymes (Winkler W. et al., 2002; Winkler W.C. et al., 2002; Winkler et al., 2003), magnesium cations (Dann et al., 2007), purines and their derivatives (Batey, 2012), second-messenger molecules (Sudarsan et al., 2008), and amino acids (Serganov and Patel, 2009). In many cases, riboswitches control the expression of genes that are involved in the biosynthesis, degradation, or transport of the respective metabolite.

Bacteria depend on the availability of amino acids for continuous protein biosynthesis. Therefore, expression of genes required for amino acid transport, synthesis, and degradation is tightly regulated. Typical sensors for amino acid levels are T-box elements and attenuators located in the mRNA. T-box RNA binds tRNAs and modulates transcription or translation of the gene under control (Grundy and Henkin, 1993). Attenuators contain codons for an effector amino acid. At a low concentration of the effector, translation stalls and formation of an anti-terminator structure is favored (Yanofsky, 1981). In addition, two riboswitches are able to bind amino acids directly. Lysine riboswitches control genes involved in lysine biosynthesis and lysine transport (Grundy et al., 2003; Rodionov et al., 2003; Sudarsan et al., 2003). Glycine riboswitches most often regulate the expression of glycine cleavage system genes, but were also found upstream of various other genes involved in the synthesis, conversion, or transport of glycine (Barrick et al., 2004). Glycine riboswitches are widely distributed and have been identified in Gram-positive and Gram-negative bacteria (Barrick and Breaker, 2007).

*Streptococcus pyogenes* [Group A streptococcus (GAS)] is a Gram-positive human pathogen responsible for a variety of diseases ranging from mild self-limiting superficial infections of the throat or skin to life-threatening invasive diseases including bacteremia and necrotizing fasciitis. Common post-streptococcal autoimmune complications are acute rheumatic fever and acute post-streptococcal glomerulonephritis. The impact of GAS diseases is especially high in resource-limited settings and in a recent publication a rise of global invasive disease burden caused by GAS was reported (Sims et al., 2016).

To limit the application and distribution of antibiotics and to identify GAS specific therapeutic targets, investigation of metabolic regulation processes can be very fruitful. Riboswitches have been already studied as promising antibacterial targets in a variety of bacteria. Modification of riboswitch function is achieved by the administration of ligand analogs, usually small compounds which are easy to manufacture and to deliver (Machtel et al., 2016). Glycine supplementation is required for optimal growth of GAS (Levering et al., 2016). The presence of a putative glycine riboswitch in GAS opens the possibility to specifically inhibit glycine metabolism or transport. Therefore, we investigated in this study gene expression regulation by a *cis*-regulatory element with sequence similarities to known glycine riboswitches. One glycine binding aptamer was predicted in the 5′-UTR of a sodium:alanine symporter family protein (SAF) gene in GAS M49 strain 591. We found that the genetic element indeed regulates glycine-dependent gene expression of the SAF gene by transcription termination/anti-termination. On a second level of regulation, SAF gene transcript stability is reduced in the presence of glycine.

## Materials and Methods

### Bacterial Strains and Culture Conditions

Group A streptococcus serotype M49 strain 591 was kindly provided by R. Lütticken (Aachen, Germany). All GAS strains were cultured in chemically defined medium (CDM) (van de Rijn and Kessler, 1980) or Todd-Hewitt broth (Thermo Fisher Scientific, Darmstadt, Germany) supplemented with 0.5% yeast extract (Thermo Fisher Scientific, Darmstadt, Germany) (THY), as indicated, at 37°C with a 5% CO_2_/20% O_2_ atmosphere. *Escherichia coli* strain DH5α (Gibco BRL, Eggenstein, Germany) was used as host for the construction, proliferation, and storage of recombinant plasmids. All *E. coli* strains were cultured in Lennox L Broth Base (Thermo Fisher Scientific, Darmstadt, Germany). For selection, antibiotics were added at the appropriate concentrations.

### Construction of Recombinant GAS Strains

Riboswitch controlled LUC reporter gene constructs were generated by cloning an 875 bp genomic fragment into the MCS of pFW11-*luc2* (Podbielski et al., 1999). Promoter controlled LUC reporter gene constructs were generated by fusing a 588 bp fragment carrying the promoter region 5′ of *ribogly* to *luc2*. Inserts were amplified by PCR using chromosomal DNA from GAS M49 strain 591 as template. All primers used for the generation of the respective fragments are listed in Supplementary Table 1. The resulting plasmids were verified by classical Sanger sequencing (GATC Biotech AG, Konstanz, Germany). GAS M49 strain 591 was transformed with the respective plasmids. Insertion of the recombinant fusion genes into the genome was verified by PCR analyses and classical Sanger sequencing (GATC Biotech AG, Konstanz, Germany) of a genomic PCR fragment.

### Quantitative Assay for Luciferase Activity

For assessment of luciferase activity from riboswitch-*luc* fusions, GAS *luc* reporter strains were grown in CDM, supplemented with 0–10 mM of the amino acids glycine, alanine, or serine as indicated. For measurement of luminescence, 1 ml aliquots of the cell suspensions were withdrawn at hourly intervals, OD_600_ was measured, and samples were processed as described by Podbielski et al. (1999). Luminescence was measured for 15 s in a Luminometer Lumimat LB 9501 (Berthold Technologies GmbH, Bad Wildbad, Germany). RLU values at each time point were calculated by subtracting luminescence at time 0 from luminescence at time x. RLU/OD_600_ were calculated to normalize for growth differences.

### Extraction of Total RNA

Group A streptococcus strains were grown for 3 h in CDM complemented with glycine as indicated. Bacteria were pelleted immediately, quickly frozen in liquid nitrogen, and stored at -80°C until use. Bacterial cells were disrupted in a homogenizer (Peqlab Biotechnologie GmbH, Erlangen, Germany). Total RNA from GAS strains was extracted according to the protocol supplied with the Direct-zol^TM^RNA MiniPrep Kit (Zymo Research, Irvine). After extraction, RNA was treated with acid phenol:chloroform:isoamyl alcohol (125:24:1), pH 4.5 (Thermo Fisher Scientific, Darmstadt, Germany), and TURBO^TM^DNAse (Thermo Fisher Scientific, Darmstadt, Germany) according to the manufacturer’s instructions. RNA was stored at -80°C until further use.

### Reverse Transcription Followed by Quantitative PCR (RT-qPCR)

Following DNAse treatment, cDNA synthesis was performed using the Superscript first-strand synthesis system for RT-PCR (Invitrogen, Thermo Fisher Scientific, Darmstadt, Germany). Quantitative PCR amplification was conducted with SYBR green (Thermo Fisher Scientific, Darmstadt, Germany) using the ViiA^TM^ 7 Real-Time PCR System (Applied Biosystems, Darmstadt, Germany). The 5S rRNA gene served as internal control. Relative expression was calculated employing the 2^-ΔΔct^ method (Schmittgen and Livak, 2008). All primers used for RT-qPCR are listed in Supplementary Table 1.

### Northern Blot Analyses

RNA samples (6 μg) were loaded onto a denaturing 1.5% formaldehyde agarose gel and separated by electrophoresis. Size standards (Millennium^TM^ RNA Marker; Thermo Fisher Scientific, Darmstadt, Germany) were loaded on the same gel. RNA was blotted onto neutral nylon membranes (Roti^®^-Nylon 0.2, Carl Roth GmbH & co KG, Karlsruhe, Germany) and UV cross-linked (UV150 mJoule/cm^2)^. Templates for the probes were generated by PCR with the primers listed in Supplementary Table 1. The 5′ end of the reverse primers contained the T7 promoter sequence (CTTAATACGACTCACTATAGGG) for *in vitro* transcription using the MEGAscript^®^ T7 Kit (Thermo Fisher Scientific, Darmstadt, Germany). Probes were labeled with biotin using the Pierce ^TM^ RNA 3′ End Biotinylation Kit (Thermo Fisher Scientific, Darmstadt, Germany). Biotin labeled RNA probes were purified using the RNA Clean & Concentrator^TM^-5 kit (Zymo Research, Freiburg, Germany). Membranes were hybridized overnight with RNA probes complementary to the respective transcripts as indicated. IRDye 800CW Streptavidin (LI-COR Biotechnology GmbH, Bad Homburg, Germany) was used for detection, and the blot was scanned on an Odyssey^®^ Imager CLX 1283 (LI-COR Biotechnology GmbH, Bad Homburg, Germany).

### Transcript Stability Determination

Group A streptococcus strains were grown for 3 h in CDM complemented with glycine as indicated. RNA synthesis was inhibited by addition of rifampicin (1 mg/ml) to the cultures. Following the addition of rifampicin, 5 ml samples were recovered after 0, 1, 2, 5, and 10 min. Samples were added to two volumes of RNAprotect Cell Reagent (Qiagen GmbH, Hilden, Germany), incubated at room temperature for 5 min, pelleted by centrifugation and quick frozen in liquid nitrogen. RNA was isolated from the samples as described above and transcript abundance was determined by RT-qPCR. The half-life of the transcripts was calculated by non-linear regression analyses (Least squares fit, GraphPad Prism).

### Statistical Analyses

All experiments were performed at least three times or as indicated by the sample size (n). Statistical significance was determined using the tests indicated in the respective figure legends.

## Results

### The Putative Glycine Riboswitch Mediates Glycine-Dependent Expression of a Luciferase Reporter Gene

**FIGURE 1:**
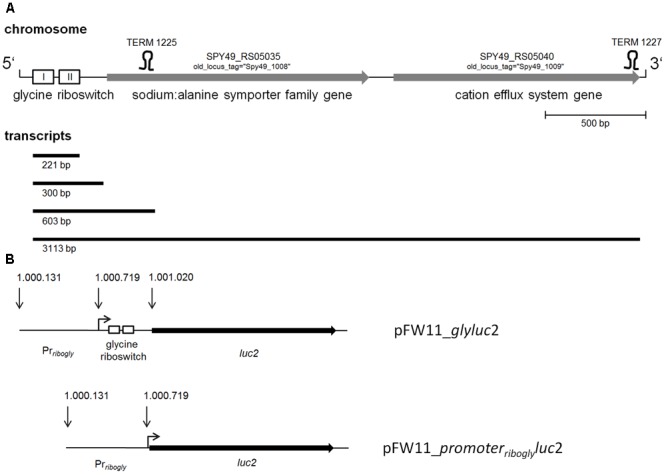
**(A)** Schematic representation of the glycine riboswitch locus. Glycine riboswitch aptamers 1 and 2 are depicted as boxes I and II; genes are depicted as gray arrows, pointing into the direction of transcription; terminators 1225 and 1227 as predicted by TransTermHP (Kingsford et al., 2007) are drawn as stem–loop structures, and transcripts detected by Northern blotting (**Figure 4**) are depicted as black lines. **(B)** Schematic representation of the riboswitch-*luc2* fusion constructs: pFW11_*glyluc*2 consists of an 889 bp genomic fragment containing the predicted glycine riboswitch sequence and its 5′ flanking genomic region including the promoter region fused to *luc2*. pFW11_*promoter_ribogly_luc*2 consists of an 588 bp fragment including the promoter region 5′ of *ribogly* directly fused to *luc2*.

**FIGURE 2:**
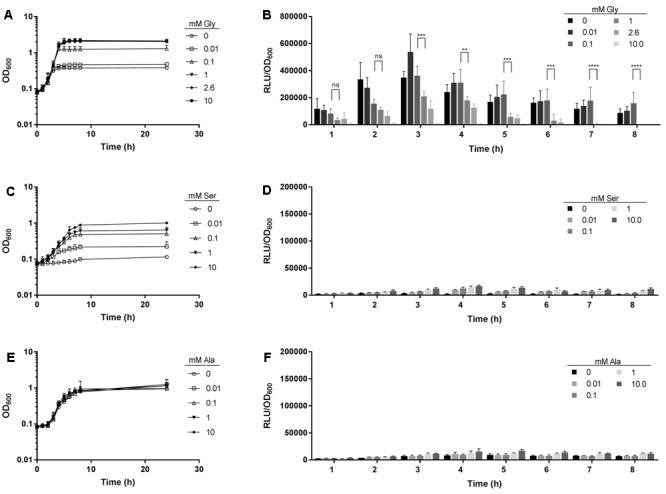
LUC reporter gene expression is repressed at high glycine concentrations. **(A)** Growth of GAS M49 strain 591/pFW11_*glyluc*2 in CDM without glycine or supplemented with different concentrations (0.01–10 mM) of glycine, *n* = 5. **(B)** Luciferase activity over time in GAS M49 strain 591/pFW11_*glyluc*2 during growth without glycine or in the presence of different concentrations of glycine, as indicated, *n* = 5. Statistical significance was determined between 0.1 and 1 mM glycine samples using the two-way ANOVA test (multiple comparisons). Differences between samples were expressed as “ns”: not significant, *P* ≥ 0.05; ^∗^*P* < 0.05; ^∗∗^*P* < 0.01; ^∗∗∗^*P* < 0.001; ^∗∗∗∗^*P* < 0.0001. **(C)** Growth of GAS M49 strain 591/pFW11_*glyluc*2 in CDM without serine or supplemented with different concentrations (0.01–10 mM) of serine, *n* = 3. **(D)** Luciferase activity over time in GAS M49 strain 591/pFW11_*glyluc*2 during growth without serine or in the presence of different concentrations of serine, as indicated, *n* = 3. **(E)** Growth of GAS M49 strain 591/pFW11_*glyluc*2 in CDM without alanine or supplemented with different concentrations (0.01–10 mM) of alanine, *n* = 3. **(F)** Luciferase activity over time in GAS M49 strain 591/pFW11_*glyluc*2 during growth without alanine or in the presence of different concentrations of alanine, as indicated, *n* = 3. Data are presented as mean values ± standard deviation.

In a previous study, we detected expression of 18 putative riboswitches using intergenic DNA tiling arrays (Patenge et al., 2012). One riboswitch candidate was identified downstream of *pcrA* (Spy49_1007c) and was designated sRNASpy491007c. It belongs to the Rfam family RF00504 of glycine riboswitches (Nawrocki et al., 2015) and is located in the 5-prime region of the sodium:alanine symporter family protein (SAF) gene (Reizer et al., 1994) (**Figure 1**). A luciferase (LUC) reporter gene system was employed to determine a potential response to glycine. An 889 bp genomic fragment containing the predicted glycine riboswitch sequence and, to allow for homologous recombination, its 5′ flanking genomic region was amplified by PCR and fused to the *luc* reporter gene carried by pFW11_*luc*2 (Podbielski et al., 1999) (**Figure 1B**). GAS M49 strain 591/pFW11_*glyluc*2 was grown in CDM containing varying concentrations of glycine. Glycine is required for optimal growth of GAS (Levering et al., 2016). Consistently, growth was poor in medium without glycine and in medium containing a low glycine concentration (0.01 mM glycine) compared to growth in medium containing 0.1 to 10 mM glycine (**Figure 2A**). LUC activity was measured over time and normalized to cell density to compensate for differences in growth (**Figure 2B**). Maximal reporter gene expression was observed following 3 h of growth in the absence of glycine and in the presence of low glycine concentrations (0.01–0.1 mM). In the presence of 10 mM glycine, reporter gene expression was always repressed. During growth in 1 mM glycine, moderate LUC activity could be detected in the exponential growth phase. Since the gene downstream of the putative glycine riboswitch encodes a putative sodium:alanine symporter and since alanine and serine are structurally similar to glycine, we tested the effect of these two amino acids on reporter gene expression. Growth of GAS M49 strain 591/pFW11_*glyluc*2 in the absence of serine and in CDM containing 0.01 mM serine was slow compared to growth in the presence of 0.1–10 mM serine (**Figure 2C**). GAS M49 strain 591/pFW11_*glyluc*2 grew similar in CDM with and without alanine (**Figure 2E**). Neither the presence of serine nor alanine did influence reporter gene expression (**Figures 2D,F**).

### The Putative Glycine Riboswitch Controls Expression the SAF Gene and a Putative Cation Efflux System Gene

**FIGURE 3:**
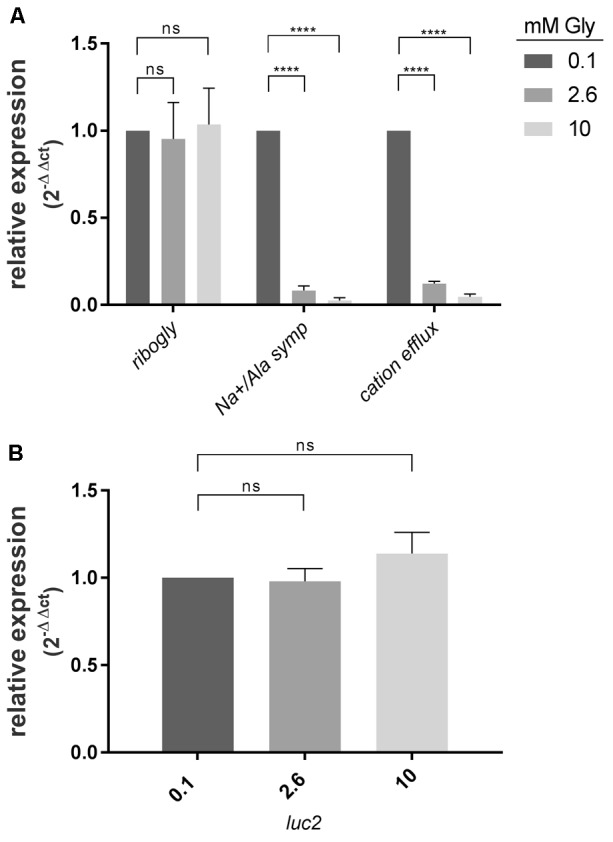
SAF gene and cation efflux system gene expression are repressed in the presence of high glycine concentrations. **(A)** Relative expression of the SAF gene (*Na+/Ala symp*) and cation efflux system gene (*cation efflux*) in comparison to glycine riboswitch (*ribogly*) transcript abundance. GAS M49 strain 591 was grown 3 h in the presence of different glycine concentrations as indicated, *n* = 3. **(B)** The promoter region does not mediate glycine-dependent induction of *luc2* expression. GAS M49 strain 591/ pFW11_*promoter_ribogly_luc*2 were grown 3 h in the presence of different glycine concentrations as indicated, *n* = 3. Relative expression Data are presented as mean values ± standard deviation. Statistical significance was determined using the Student’s *t*-test. Differences between samples were expressed as “ns”: not significant, *P* ≥ 0.05 and ^∗∗∗∗^*P* < 0.0001.

To address the question, whether the putative glycine riboswitch controls expression of its downstream genes, RT-qPCR experiments were conducted. WT bacteria were grown in CDM containing 0.1, 2.6, and 10 mM glycine. Total RNA was isolated, reverse transcribed, and amplified using primers specific for the putative glycine riboswitch (*ribogly*), the SAF gene (*Na^+^/Ala symp*), and the putative cation efflux system gene (*cation efflux*), respectively (**Figure 3A**). Transcript abundance at 0.1 mM glycine served as calibrator for all three reactions. Abundance of the riboswitch RNA was unaltered at all glycine concentrations tested. In contrast, the SAF and the cation efflux system gene mRNA were dramatically decreased at 2.6 and 10 mM glycine in comparison to 0.1 mM glycine.

### Glycine-Dependent Gene Control Is Not Based on Changes of Promoter Activity

To investigate the regulatory influence of the promoter region, a 588 bp fragment carrying the promoter region 5′ of *ribogly* was fused to *luc* (**Figure 1B**). GAS M49 strain 591 was transformed with pFW11_*promoter_ribogly_luc*2. Transcript abundance of *luc2* was determined by RT-qPCR following growth in the presence of 0.1, 2.6, and 10 mM glycine. Transcript level at 0.1 mM glycine served as calibrator. Expression of *luc2* was not influenced by the glycine concentration in the medium (**Figure 3B**).

### Transcript Sizes Are Dependent on the Glycine Concentration in the Medium

**FIGURE 4:**
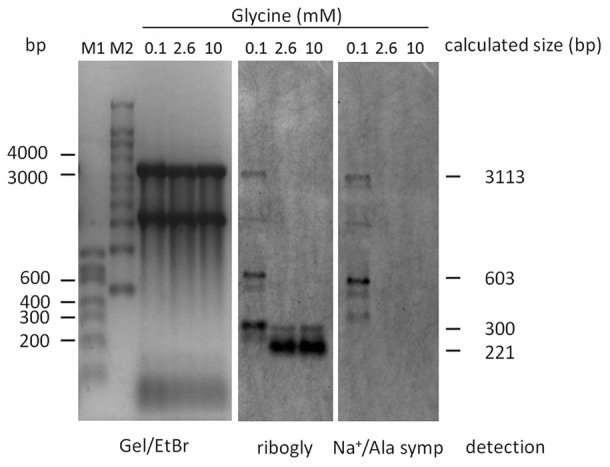
Northern blot analyses. RNA was extracted from bacteria grown in 0.1, 2.6, and 10 mM glycine as indicated. RNAs were separated by denaturing agarose gel electrophoreses and subsequently blotted onto a nylon membrane. The membrane was hybridized with probes specific for the glycine riboswitch (*ribogly*), stripped, and hybridized with a probe specific for the SAF gene (Na+/Ala symp). The ethidium bromide stained gel served as loading control.

Expression of both, the SAF gene and the cation efflux system gene, was repressed in the presence of high glycine concentrations (**Figure 3**). Primers to amplify gene junctions were designed and used in reverse-transcriptase experiments followed by PCR. The detection of a PCR product suggested co-transcription of the two genes. To verify this result, Northern blot analyses were performed. WT bacteria were grown in CDM containing 0.1, 2.6, or 10 mM glycine, respectively. Total RNA was isolated from the cultures and separated by agarose gel electrophoresis. Blots were sequentially hybridized with RNA probes specific for *ribogly* and the SAF gene transcript (*Na^+^/Ala symp*). In the presence of high glycine concentrations (2.6 and 10 mM), the *ribogly* probe detected a highly abundant 200 bp band, which might represent a transcription termination or a RNA cleavage product (**Figure 4**). No SAF gene transcript could be detected (**Figure 4**). Consistent with the *luc* reporter gene assay and RT-qPCR results, expression of the gene was repressed under these conditions. In the presence of 0.1 mM glycine, a 3 kb band could be detected with the *ribogly* and the *Na+/Ala symp* probes. The apparent size of the band corresponds to the calculated size of a co-transcript of the SAF gene and the cation efflux system gene (3113 bp, **Figure 1**). A second band with the apparent size of 600 bp could be detected with both probes. This corresponds to the product of termination within the SAF gene at the putative terminator TERM 125 (TransTermHP v2.07) (Kingsford et al., 2007). A 300 bp band was detected using the *ribogly* probe but not following hybridization with the *Na+/Ala symp* probe. The transcripts detected by Northern blotting are depicted schematically in relation to the corresponding genes (**Figure 1**).

### SAF Gene Transcript Stability Is Reduced at High Glycine Concentrations in the Medium

**FIGURE 5:**
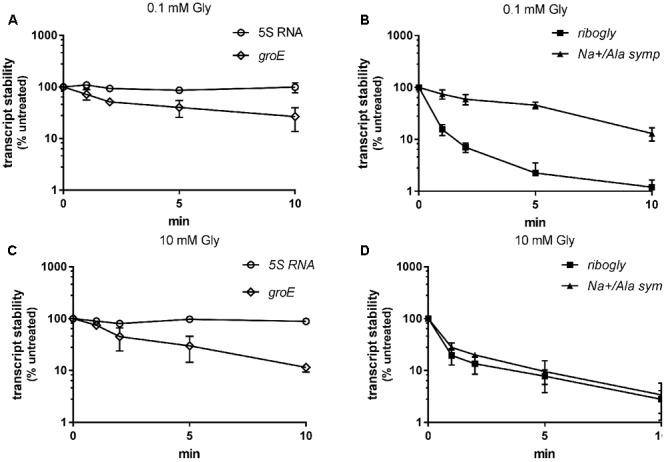
Transcript stability under low (0.1 mM) and high (10 mM) glycine conditions. Stability of the glycine riboswitch (*ribogly*) and the SAF gene (*Na+/Ala symp*) as determined by RT-qPCR following treatment of the culture with rifampicin, *n* = 3. 5S rRNA and *groES* mRNA served as controls. Stability is presented as percent transcript level relative to time-point zero. **(A)** 5S rRNA and *groES* mRNA stability at 0.1 mM glycine. **(B)** *ribogly* and *Na+/Ala symp* mRNA stability at 0.1 mM glycine. **(C)** 5S rRNA and *groES* mRNA stability at 10 mM glycine. **(D)** *ribogly* and *Na+/Ala symp* mRNA stability at 10 mM glycine. The data are presented as the mean values ± standard deviation.

Transcripts of genes controlled by riboswitches are often processed or exhibit differential stability (Mellin and Cossart, 2015). We aimed to investigate *ribogly* and SAF gene transcript stability under repressing and non-repressing conditions. WT GAS M49 strain 591 was grown in the presence of 0.1 or 10 mM glycine, respectively. Transcription was stopped by addition of rifampicin and samples were collected for RT-qPCR analyses at 1, 2, 5, and 10 min following rifampicin treatment (**Figure 5**). 5S rRNA and *groES* mRNA served as controls. 5S rRNA was stable over the observation period. The half-life of *groES* mRNA was similar during growth in 0.1 mM glycine (1.8 min) and 10 mM glycine (1.9 min). Stability of *ribogly* was low under both conditions. Its half-life was 0.4 min in 0.1 and 10 mM glycine. In contrast, the stability of *Na+/Ala symp* was low during growth in 10 mM glycine (0.5 min) but much higher in 0.1 mM glycine (5 min), suggesting an additional ribonuclease-dependent regulation level.

### *ribogly* Belongs to the Tandem Aptamer Glycine Riboswitches

**FIGURE 6:**
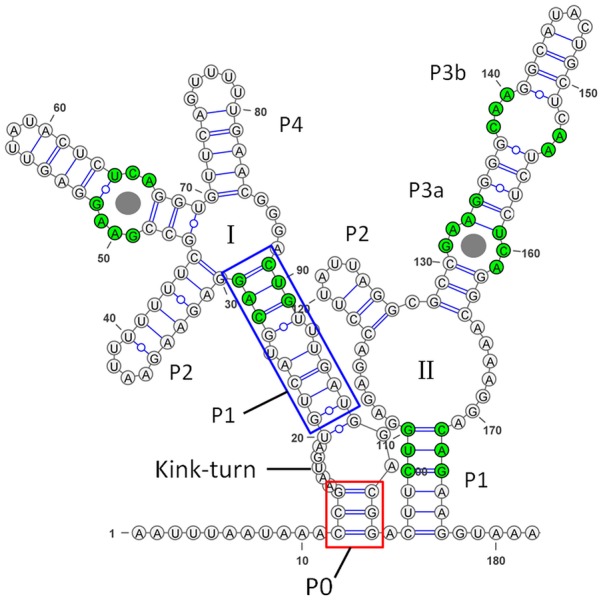
Secondary structure of the glycine riboswitch in *Streptococcus pyogenes*. The secondary structure was predicted using RNAfold and illustrated using VARNA GUI (Darty et al., 2009). Conserved residues are highlighted in green (Ruff et al., 2016). Glycine binding sites are indicated by gray circles. P1, the Kink-turn motif, and P0 are highlighted following the observations from the glycine riboswitch in *Vibrio cholerae* (Esquiaqui et al., 2014).

In the reference genome of *S. pyogenes* NZ131 (accession number: NC_011375.1), a 90 bp putative glycine riboswitch was annotated. A search using the rfam database (Nawrocki et al., 2015) predicted the presence of one glycine-binding aptamer. Since we observed a band of approximate 200 bp in Northern blot analyses of total RNA from GAS M49 strain 591 (**Figure 4**), we performed secondary structure prediction using RNAfold (The ViennaRNA Web Services^1^). Secondary structure modeling was based on 182 bp of the reference genome of *S. pyogenes* NZ131. The result is illustrated in **Figure 6** using VARNA GUI (Darty et al., 2009). The analysis revealed the typical consensus structure of a tandem aptamer riboswitch containing two glycine binding sites (Esquiaqui et al., 2014; Ruff et al., 2016). The conserved residues are labeled in green.

## Discussion

Riboswitches often regulate expression of genes involved in the metabolism or transport of their ligands. Typically, glycine riboswitches induce the expression of genes responsible for glycine cleavage or export following glycine binding (Barrick et al., 2004). In *Bacillus subtilis*, the glycine riboswitch is composed of two similar aptamers followed by a single expression platform (Mandal et al., 2004). In *S. pyogenes*, one aptamer, similar to the *B. subtilis* aptamers, was predicted upstream of a putative SAF gene. Previously, we observed expression of the riboswitch region in GAS M49 strain 591 by intergenic tiling arrays (Patenge et al., 2012). In the SF370 clinical isolate, expression of the putative streptococcal riboswitch was detected by Northern blot analyses (Le Rhun et al., 2015). In this work, we investigated whether the putative glycine riboswitch mediates glycine-dependent gene regulation.

LUC reporter gene assays and Northern blot analyses revealed that expression of the SAF gene was repressed in the presence of glycine. The corresponding promoter region did not mediate glycine-dependent downregulation of *luc* in a promoter–reporter gene fusion construct. As a general rule, amino acid riboswitches increase the expression of glycine cleavage system genes or glycine export protein genes upon binding to glycine (Serganov and Patel, 2009; Serganov and Nudler, 2013). In *Streptomyces griseus* a detoxification system is activated by a glycine riboswitch (Tezuka and Ohnishi, 2014). In contrast, *lysC* in *B. subtilis* is repressed by a lysine-responsive riboswitch due to transcription termination in the presence of a high L-lysine concentration (Phan and Schumann, 2009). The *lysC* gene of *B. subtilis* consists of two overlapping reading frames coding for the α- and β-subunits of a lysine-responsive aspartokinase II, which catalyzes the first step in the biosynthesis of methionine, lysine, and threonine (Chen et al., 1987). The SAF gene in *S. pyogenes* encodes a member of the Alanine or Glycine:Cation Symporter (AGCS) Family [Transporter Classification Database (TCDB)] (Saier et al., 2016). Proteins belonging to the AGCS family have been reported to transport alanine and/or glycine in symport with Na^+^ and/or H^+^. The *dagA* gene from the marine bacterium *Alteromonas haloplanktis* is a sodium-dependent transporter and is involved in the uptake of glycine and glutamine (MacLeod and MacLeod, 1992). We hypothesize that binding of glycine by the riboswitch in *S. pyogenes* leads to the downregulation of a so far unidentified glycine uptake system.

The truncated form of the *B. subtilis* riboswitch RNA controlling *lysC* is predominantly produced, even under conditions promoting anti-termination (Phan and Schumann, 2009). In contrast, we did only detect small amounts of the truncated 200 bp riboswitch RNA under inducing conditions. Stability of the riboswitch RNA was low at all glycine concentrations tested. The half-life of the full-length transcript decreased at high glycine concentrations. A combination of classical riboswitch function and mRNA stability control has been reported recently. In *E. coli, lysC* expression is controlled on the level of translation initiation by a lysine riboswitch. Upon lysine binding, the riboswitch adopts a conformation that sequesters the ribosomal binding site and at the same time exposes RNaseE cleavage sites (Caron et al., 2012). RNAse E is a component of the RNA degradosome in Gram negative bacteria (Callaghan et al., 2004). In most Gram positive organisms, RNaseE is replaced by other ribonucleases, e.g., RNaseY and RNases J1 and J2 (Cho, 2017). In *S. pyogenes*, RNAse Y is involved in mRNA turnover (Chen et al., 2013). Structural investigation of the GlmS ribozyme from *B. anthracis* revealed autocatalytic cleavage of GlmS mRNA at a single site 5′ of the riboswitch sequence following binding of glucosamine-6-phosphate (GlcN6P). Specific cleavage renders GlmS mRNA susceptible to degradation by RNase J (Cochrane et al., 2007). It is tempting to speculate that processing of the glycine riboswitch controlled transcript in *S. pyogenes* leads to the exposure of RNase cleavage sites and thereby to decreasing SAF gene transcript stability.

The glycine riboswitch is the only riboswitch that exhibits tandem architecture, with two adjacent, homologous aptamers followed by a single expression platform. In *B. subtilis*, glycine binding by the riboswitch aptamers has been initially reported to function in a cooperative manner (Mandal et al., 2004). However, a full-length derivative of the riboswitch containing its extended 5′ leader, did not show cooperative binding (Sherman et al., 2012). The tandem glycine riboswitch from *Vibrio cholerae*, including the leader sequence, was studied using an equilibrium dialysis-based assay. The results showed that ligand binding by aptamer-1 is linked to aptamer dimerization and stabilizes the P1 stem of aptamer-2, which controls the expression platform (Ruff and Strobel, 2014). In a recent study, analysis of sequenced genomes revealed a significant number of singlet glycine riboswitches. Several singlet riboswitches were characterized biochemically and it could be demonstrated that singlet riboswitches were able to bind glycine with affinities comparable to those of previously published tandem glycine riboswitches. Conserved stem–loop structures (ghost aptamers), situated up- or down-stream of the singlet aptamer, respectively, form interactions with the aptamer domain that are necessary for ligand-binding activity (Ruff et al., 2016). In *S. pyogenes*, one aptamer was annotated upstream of the SAF gene. A second aptamer containing a P4 stem was revealed by *in silico* secondary structure predictions. The sequence directly upstream of the first aptamer is similar to the conserved leader region, which interacts with the recently discovered K-turn linker of tandem glycine riboswitches and modulates ligand binding (Kladwang et al., 2012; Sherman et al., 2012; Baird and Ferre-D’Amare, 2013; Esquiaqui et al., 2016). This indicates that the *S. pyogenes* glycine riboswitch belongs to the class of tandem riboswitches, featuring the recently identified K-turn linker.

Inducible expression systems are useful tools in molecular biology. Characterization of essential genes and production of potential toxic gene products are only two of many examples for the requirement of conditional gene expression control. Riboswitch based systems for precise gene regulation have the advantage, that expression can be controlled by the addition of small compounds that easily enter the cell and that are in many cases comparably inexpensive (Machtel et al., 2016). A glycine-inducible expression system has been developed using the riboswitch from *B. subtilis*. The authors could demonstrate glycine-dependent production of recombinant proteins (Phan and Schumann, 2007). With a comparable strategy, a gene knock-down or knock-out system could be engineered, employing the *S. pyogenes* glycine riboswitch.

Riboswitches are potential targets for antimicrobial therapies (Machtel et al., 2016). Extensively studied targets include purine riboswitches. Purines are essential for bacterial survival and purine riboswitches control purine metabolism and transport (Lunse et al., 2014). There is an ongoing search for suitable purine analogs that bind the riboswitch with comparable affinity as guanosine (Kim et al., 2009; Mulhbacher et al., 2010). Beside essential pathways, riboswitch control of biofilm formation is a promising target for antimicrobial drugs (Reyes-Darias and Krell, 2017). Lysine riboswitches have also been explored as potential targets and several lysine analogs with high affinity binding have been identified, but there were difficulties arising from toxicity of the compounds and bacterial resistance (Blount et al., 2007). Glycine is required for optimal *S. pyogenes* growth. The *S. pyogenes* glycine riboswitch is highly conserved among different serotypes. Sequence identity of the riboswitch within the completed *S. pyogenes* genomic sequences is 99–100%. Downregulation of streptococcal glycine transport by targeting the glycine riboswitch with a glycine analog could serve as a novel therapeutic strategy. Alternatively, ligands for the not yet characterized riboswitches in *S. pyogenes* should be identified and investigated for their drug target potential.

## Author Contributions

AK and NIP performed the experiments presented in the manuscript. AK, NIP, BK, and NAP contributed to the design of this study, analyses, and interpretation of the data, drafting the manuscript, and approved it for publication.

## Conflict of Interest Statement

The authors declare that the research was conducted in the absence of any commercial or financial relationships that could be construed as a potential conflict of interest.

## Footnotes

1 http://rna.tbi.univie.ac.at/
