# Supplementary material for: A Glycine Riboswitch in Streptococcus pyogenes Controls Expression of a Sodium:Alanine Symporter Family Protein Gene
Source: Front Microbiol. 2018 Feb 20;9:200. doi: 10.3389/fmicb.2018.00200 (PMC5829553; doi:10.3389/fmicb.2018.00200)
Supplement: Supplementary file 1 [file Table_1.DOCX]

Supplementary Material

**A Glycine Riboswitch in *Streptococcus pyogenes* controls Expression of a Sodium:Alanine Symporter Family Protein Gene**

Afsaneh Khani, Nicole Popp, Bernd Kreikemeyer, and Nadja Patenge*

*** Correspondence:** Nadja Patenge: nadja.patenge@med.uni-rostock.de

| Supplementary Table1: Sequences of primers used for PCR | | |
| --- | --- | --- |
| Primer Name | Forward Strand (5'->3') | Reverse Strand (5'->3') |
| Sequences of primers used for vector construction | | |
| *ribogly*-*luc* | TGCGTCGACATGCGCTTCATCAACGTTCG | CCTCGAGCTCCTAAAAGTCATCGAAGACG |
| *promoter_ribogly_* | TCGGATCCATGCGCTTCATCAACG | CCTCGAGAATTAGGACTATCATACCTC |
| Sequences of primers used for RT-qPCR | | |
| 5S RNA | AGCGACTACCTTATCTCACAG | GAGATACACCTGTACCCATG |
| RT_*ribogly* | GCATACTGCTCAATCTCTCAGGC | CTCCTAAAAGTCATCGAAGACGCC |
| RT_*luc* | GAGACATAGCTTACTGGGACG | TATCGACTCCAATTCAGCGGG |
| *Na+/Ala symp* | AATCAGCCTTCACTCCGACAGC | ACTCATTGGAGAAGACACCGCG |
| *cation efflux* | GTCTCAACGAGCTAGGACC | GGCAGGCTCAACGTGAATG |
| *Ribogly*_*Na+/Ala symp* | TCAGGCAAAAGGACAGAAGG | CGAGTGCTATCATCTTTCTCTCC |
| *Na+/Ala symp_ cation efflux* | CGCCAGTTGTTATTTTAGAAACC | GATTGGCATCGGCTGGTTGGC |
| RT_*groES* | TCGGGTGTTCGCACTATTACAG | CATGCCCATTTTCAACTAAAACC |
| Sequences of primers used for Northern blot probes | | |
| *ribogly* | GCATACTGCTCAATCTCTCAGGC | cttaatacgactcactataggCTCCTAAAAGTCATCGAAGACGCC |
| *Na+/Ala symp* | AATCAGCCTTCACTCCGACAGC | cttaatacgactcactataggACTCATTGGAGAAGACACCGCG |
